# Making Molecular Diagnostics Faster

**DOI:** 10.1111/ijlh.14487

**Published:** 2025-04-22

**Authors:** Carl T. Wittwer, Luming Zhou, Felix Ye, Adam Millington, Adrian de Cola, Noriko Kusukawa

**Affiliations:** ^1^ Crestwood Technology Camden Maine USA; ^2^ Department of Pathology University of Utah Salt Lake City Utah USA

**Keywords:** alkaline lysis, extreme PCR, high‐speed DNA melting, molecular diagnostics, point‐of‐care testing

## Abstract

**Background:**

Over the past 40 years, molecular diagnostic methods have evolved from multi‐step, time‐consuming protocols towards either rapid targeted tests or expansive, massively parallel testing.

**Aims:**

Here we consider the speed limits of targeted molecular diagnostics, considering the three sequential required steps: nucleic acid preparation, amplification, and analysis.

**Materials & Methods:**

Instead of the bind/wash/elute steps commonly used for nucleic acid extraction, simple alkaline lysis of whole blood results in a suspension ready for PCR in seconds that can be added directly to an appropriately buffered PCR master mix. For amplification, the time requirements of PCR are typically limited by the temperature cycling instrumentation and not by biochemistry.

**Results & Discussion:**

By lowering sample volumes, increasing the surface area to volume ratio, decreasing the thickness of the sample container, decreasing the amplicon size, and inducing rapid temperature changes by a myriad of innovative means, 30 cycles of PCR can easily be completed in less than 5 min. By increasing primer and polymerase concentrations in synchrony with even faster cycling (< 2 s cycles), “extreme PCR” has amplified a 60 bp human genomic target in < 15 s (35 cycles) with high yield and specificity. For analysis, cumbersome, contamination‐prone gel analysis can be replaced by melting curve analysis. Although melting curve analysis usually takes up to an hour on commercial instrumentation, precise temperature control can enable single base genotyping in 1–4 s.

**Conclusion:**

These advances demonstrate the feasibility of sample‐to‐answer molecular diagnostics in seconds.

## 1 Introduction

Since the discovery of the structure of DNA in 1953 [1], we have continued to create ways to interrogate this information‐rich molecule. Information contained in overall cellular DNA content, individual chromosomes, chromosome bands, copy number alterations, and sequence variants are all important in health and disease. The sequencing of the human genome is heralded as a great accomplishment of the 21st century, yet the subsequent reduction in sequencing cost and time to results for whole genome analysis is perhaps more impressive. In contrast, despite widespread interest in point‐of‐care diagnostics, progress towards providing inexpensive, rapid, routine diagnostics has been slow. Indeed, the COVID19 pandemic reminded us that availability and turnaround times of simple molecular tests can be crucially important. In this article, we focus on shortening the time‐to‐result of targeted molecular testing.

Advantages of making molecular diagnostics faster include reduced disease spread, timely treatment and triage, and better utilization of health care resources. The value of point‐of‐care testing depends on how quickly the results are available. If results are delayed by an hour or even 5 min, the risk of losing contact with the patient increases. Treatment at the time of testing reduces preanalytical and postanalytical errors. Accelerating molecular diagnostics improves patient care, enhances public health, and increases the capacity of the health care system.

To shorten the time of targeted molecular diagnostics, we'll use real‐time PCR and DNA melting analysis. Although isothermal methods of amplification [2] simplify instrument requirements and can be completed in 3–20 min, extreme PCR and high‐speed melting can be completed in seconds. Much of what is reviewed here has been published before, although the integration of alkaline sample preparation directly into PCR is new. The methods and instruments described here are not currently available commercially, but they are based on elementary principles that are easy to understand. There will come a time when molecular diagnostics, “while you wait,” becomes a reality. In that scenario, every second counts.

The three stages of any molecular test are: (1) sample preparation, (2) amplification, and (3) analysis. Minimizing time to result requires that all stages are rapid; if the sample preparation takes an hour, it does not matter if PCR and analysis can be completed in seconds.

## 2 Sample Preparation

Conventional sample preparation involves extraction, purification, and quantification of DNA or RNA before amplification [3]. Typical steps involve cell lysis, binding of the nucleic acid to a solid support, washing contaminants from the matrix, and final elution of the nucleic acid that can be checked for purity and its concentration determined. However, the multiple steps required reduce sample yield, and even with automation the time required is typically 15–60 min. On the other hand, some samples are PCR compatible without any processing, and the initial heat denaturation step of PCR is adequate to make DNA available for PCR. Extraction‐free methods of preparing clinical samples for PCR amplification became popular during the COVID19 pandemic, where SARS‐CoV‐2 was directly detected in saliva with minimal sample preparation [4]. However, it is much easier to liberate the nucleic acid from viruses or bacteria than eukaryotic DNA that is densely bound by chromatin. Some methods isolate both DNA and RNA; others are designed to only isolate one or the other. We consider here the rapid liberation of DNA from blood for PCR amplification.

### 2.1 Alkaline Lysis as a Method for DNA Sample Preparation

Alkaline lysis with sodium hydroxide (NaOH) is a standard procedure to prepare plasmid DNA from bacteria [5]. Alkaline lysis for preparing human genomic DNA from blood, epithelial cells, and semen has also been reported [6] in which 5 μL of human blood was added to 20 μL 0.2 N NaOH for 1–5 min at room temperature and neutralized with 180 μL 40 mM Tris, pH 7.5 for a DNA yield of 65%. Five μL of the extract was then added to a 50 μL PCR mixture.

**FIGURE 1:**
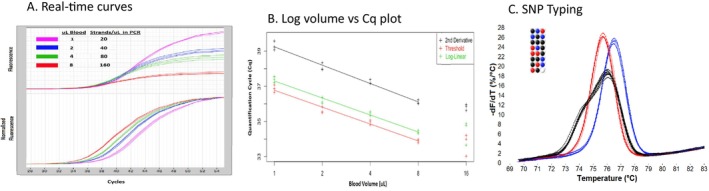
Performance of NaOH‐treated whole blood in PCR. (A) Real‐time PCR. Fresh whole blood (1–8 μL) was added to 200 mM NaOH for a total volume of 100 μL, mixed for 5 s, and 1 μL added with agitation into a 9 μL PCR master mix. The PCR included 0.5 μM each primer (*CFTR* exon 11, forward primer TGTGCCTTTCAAATTCAGATTG, reverse primer CAGCAAATGCTTGCTAGACC, amplifying a 202 bp product), 200 μM each dNTP, 0.04 U/μL KlenTaq, 3 mM Mg(Cl)_2_, 250 μg/mL BSA, 1× LCGreen Plus dye, and 50 mM Tris (master mix pH = 8.0, pH after lysate addition 8.7). Samples were amplified on a capillary LightCycler with an initial 30 s denaturation at 95°C, followed by 55 cycles of 95°C for 0 s, 55°C for 0 s, and 72°C for 5 s. The maximum relative fluorescence (top) decreased with blood volume suggesting fluorescence quenching, but normalization to the final fluorescence (bottom) revealed that the PCR efficiency was not affected with equal spacing between 2‐fold dilutions. (B) Log volume vs. quantification cycle (Cq) plot of the curves shown in panel A using three different methods of Cq determination [7]. The curve slopes indicated a PCR efficiency of 100% within the confidence interval. Higher volumes of blood (16 μL) did show PCR inhibition. (C) Example of SNP genotyping [8] using NaOH‐lysed whole blood. The protocol used 4 μL of whole blood, amplifying a 76 bp fragment surrounding an A/G SNP (rs1024116) with 0.5 μM primers CCATGTGTTCTAATAAAAAGGATTGC and TGGGAAGTGAGCAAAAGTAAATACA. After PCR, a high‐resolution melting curve was obtained at 0.3°C/s. Homozygous A samples are shown in red, homozygous G samples in blue, and heterozygous AG samples in black.

We modified this procedure to shorten the time and allow for direct addition of the alkaline lysate into an appropriately buffered PCR solution. Unless specified otherwise, 4 μL of freshly drawn human blood was added to 96 μL of 200 mM NaOH with mixing for 5 s at room temperature. Then, 1 μL of the extract was directly added to a 9 μL PCR master mix with agitation that was buffered with 56 mM Tris, pH 8.0. The resulting solution was 50 mM in Tris, pH 8.7 (calculated by the Henderson–Hasselbach equation and ignoring any buffering capacity of blood) and was PCR compatible. The NaOH lysis was rapid and resulted in PCR‐compatible DNA in high yield. By real‐time PCR, the Cq changed less than 1 cycle as the time in NaOH was shortened from 60 to 5 s (the fastest time we could manually process the sample). By limiting dilution analysis, the yield of DNA strands available for amplification based on the white blood cell count was 84%–146% with a 95% confidence interval. The performance of NaOH‐treated blood in PCR is shown in Figure 1 and details for the reaction are in the legend. As the dosage of blood increases, there is relative quenching of fluorescence (Figure 1A, top), but normalizing the curves to the endpoint fluorescence shows the expected equal spacing of 2‐fold dilutions (Figure 1A, bottom). PCR efficiency, as calculated from the log of the blood volume processed vs. quantification cycle (Cq) graph, was 100% (within error limits), although at higher concentrations (16 μL blood), PCR efficiency does decrease (Figure 1B). DNA prepared by alkaline lysis of blood can be used in sensitive downstream applications, such as single base genotyping by high resolution melting (Figure 1C).

DNA (unlike RNA) is stable in basic solution. It is surprising that alkaline lysis is not used more often to prepare DNA for PCR from biological samples. Because the lysate can be added directly into PCR, it provides a simple and rapid preamble to amplification.

## 3 Amplification

Before PCR, gene cloning required days to weeks of biological growth and biochemical purification. PCR introduced “cell free molecular cloning” that required only 3–4 h [9]. With the advent of heat stable polymerases and instruments to drive the process forward by repetitively changing the temperature for template denaturation, primer annealing, and enzymatic extension, over a million‐fold amplification of targeted sequences was achieved without external processing. However, the time requirements were limited by the available instrumentation, and it soon became clear that PCR could be performed much faster, simply by decreasing the transition time between temperatures, and minimizing the holding times, particularly at the primer annealing and product denaturation stages.

### 3.1 Rapid Cycle PCR

The “Rapid Cycler”, introduced by Idaho Technology in 1990, produced enough DNA of a 536 bp target of human genomic DNA in 10 min to be observed on agarose electrophoresis gels [10]. The instrument used circulating hot air for temperature control of small (10 μL) samples in capillary tubes. Temperature control of the sample was homogeneous and precise enough that programmed denaturation and annealing temperatures did not need any extra time for the bulk solutions to reach temperature. Once the temperature was attained, the next transition to the subsequent temperature was triggered. That is, 0 s holds were employed for denaturation and annealing. Experimentally, it was shown that product yield was maximal with 0 s denaturation holds during cycling [11]. Furthermore, rapid transitions to the annealing temperature, and 0 s annealing holds resulted in the best amplification specificity. At the primer concentrations used (0.5 μM), annealing occurred very quickly, not requiring any holding time at the annealing temperature. Faster PCR was better PCR in terms of specificity. However, limiting the extension time too far did lead to a loss of product yield. Chain extension by the polymerase takes time, and the required time depends on the length of the PCR product and the speed of the polymerase.

### 3.2 The Race for Faster PCR Speeds

Rapid cycle PCR, first introduced by the Rapid Cycler, catalyzed a race toward faster and faster speeds for completion of PCR. Many systems developed between 1990 and 2015 were able to attain between 10 and 20 s cycles, validating and improving on the rapid cycle claim of 20 s cycles (30 cycles in 10 min). Of particular interest are published articles with cycle times of 10 s or less (30 cycles in less than 5 min), and those are compiled in Table S1. Thirty‐four articles with cycle times of 10 s or less are included and compared to the original 20 s rapid cycle PCR publication. Articles are classified according to cycle time, instrument design, heating and cooling methods, identity of the template and its initial concentration, product length, polymerase identity, polymerase concentration, primer concentration, whether a negative control was included, product yield, publication year, and a hyperlink to the publication. Many creative and interesting ways to rapidly temperature cycle samples have been published, including radiative heating of suspended nanoparticles and conduction/convection by forced air, pressurized gas, water, or gallium. In some systems, the sample is stationary, and in others, it is transferred between heat sinks, either by moving the sample container or just the sample. Continuous flow PCR on a chip was introduced in 1998 with the sample flowing between copper blocks at different temperatures [12]. Ten million copies/μL of a 1000 bp PCR product was used as a template to amplify a 176 bp product. As the cycle times decreased, so did the product yield, but a faint gel band was observed with 6.6 s cycles. Different studies used different templates, ranging from (least to most complex): PCR products, plasmids, virus, phage, bacteria, or eukaryotic genomic DNA, and their initial concentrations varied widely between 1.8 × 10^8^ and 10^−3^ copies/μL (the lowest concentrations analysed by digital PCR [13, 14]). Product lengths varied from 60 to 500 bp and a variety of different polymerases were used at concentrations from 1 to 8000 nM. Primer concentrations varied between 360 and 20,000 nM. Some studies did not include no template controls, and others claimed successful results if the positive target fluorescence was greater than that of the no template control. Although some studies could document high PCR efficiency and good yield, many showed very dim bands on gels, suggesting low yields. In general, many studies used high concentrations of low complexity templates, and the PCR yield appeared compromised at the fastest speeds. In contrast to this trend, some reports with the fastest cycle times retained high efficiency and yield by using high concentrations of primers and polymerase, a development known as “extreme PCR”

### 3.3 Extreme PCR

It was clear from rapid cycle PCR that denaturation is a very fast process. As a first order reaction, the rate of denaturation depends on the temperature and is directly proportional to product concentration. The fraction of product denatured at a given time and temperature is constant. Primer annealing, however, is a second order reaction. It depends not only on temperature, but on both available single stranded product and primer concentrations. However, the fraction of product annealed at any given time and temperature is directly proportional to the primer concentration. Hence, by increasing the primer concentration, you increase the rate of annealing. If temperature cycling is fast enough that limited primer annealing lowers PCR efficiency, increasing the primer concentration will increase the PCR efficiency. With regard to the polymerase, strand extension is a complex process involving the polymerase, template, dNTPs, and Mg^++^. To the extent that polymerase extension is limited by polymerase binding, extension rates could be increased by increasing the polymerase concentration. Extreme PCR combines very rapid temperature cycling (< 2 s cycles) with high primer and polymerase concentrations to achieve efficient PCR amplification.

**FIGURE 2:**
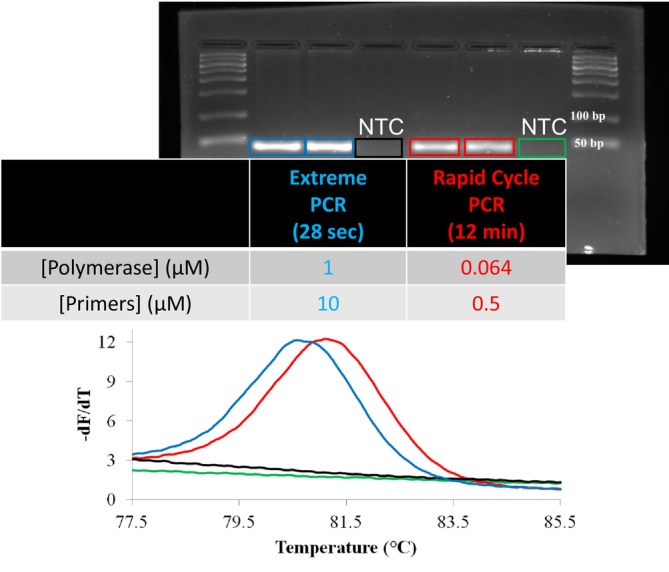
Extreme PCR compared to rapid cycle PCR [15]. PCR was performed in 5 μL volumes with 50 mM Tris, pH 8.3, 3 mM Mg(Cl)_2_, 200 μM each dNTP, 500 μg/mL nonacetylated BSA, 2% glycerol, and 50 ng purified human genomic DNA of the GG genotype. The *KCNE1* primers were CCCATTCAACGTCTACATCGAGTC and TCCTTCTCTTGCCAGGCAT, surrounding a G > A variant (rs#1805128) and were present at either 0.5 μM (rapid) or 10 μM (extreme). KlenTaq DNA polymerase was included at either 0.064 μM (rapid) or 1.0 μM (extreme). Two‐temperature cycling between 50°C and 90°C at maximal rates without temperature holds were used in both cases. Even though the cycling times differed by 25‐fold, specificity and yield as demonstrated on agarose gels and melting analysis were similar. The left shift of the melting curve after extreme amplification can be explained by the higher glycerol content contributed by a greater volume of polymerase. No template controls (NTC) showed no amplification. This figure is modified from a prior publication [15].

Typical primer and polymerase concentrations in extreme PCR are greater than 10‐fold that of normal PCR. Under conventional temperature cycling conditions, these concentrations would result in non‐specific products. However, with extreme temperature cycling (< 2 s cycles), specific products with good yield are observed [15]. Both increased primer and polymerase concentrations are required for amplification by extreme PCR. Figure 2 shows gel and melting curve analysis of the PCR products from the amplification of a 45 bp fragment of human genomic DNA in a 5 μL reaction under conditions of rapid cycling and extreme PCR. Polymerase and primer concentrations in extreme PCR were about 20‐fold higher than those used for rapid cycle PCR. Extreme PCR was completed in 28 s, about 25‐fold faster than rapid cycle PCR, which took 12 min. Specific gel bands were observed for both protocols, and no template controls (NTC) were negative. Extreme PCR did not compromise the observed yield; the gel bands from extreme PCR even appeared stronger than those from rapid cycle PCR.

**FIGURE 3:**
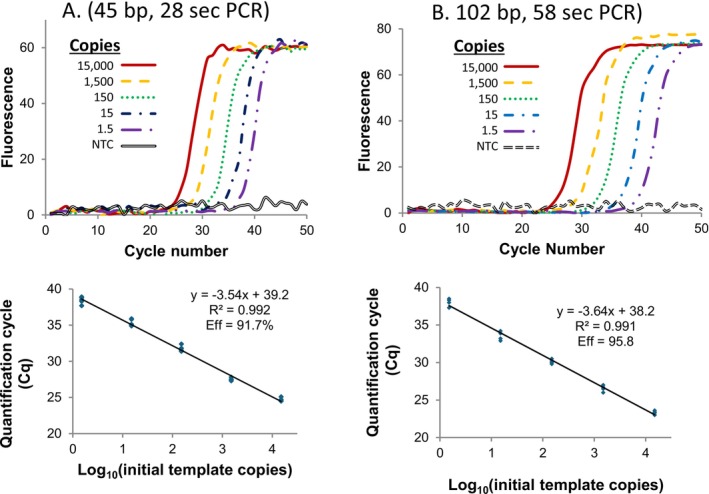
Sensitivity and PCR efficiency of extreme PCR [15]. In panel A, the 45 bp *KCNE1* fragment was amplified as in Figure 2 except that the template concentration varied from 1.5 to 15 000 copies. In panel B, a 102 bp fragment surrounding an *NQ01* variant (rs#1800566) was amplified with primers CTCTGTGCTTTCTGTATCCTCAGAGTGGCATTCT and CGTCTGCTGGAGTGTGCCCAATGCTATA, similarly varying the template concentration. For *NQ01*, temperature cycling was between 92°C (no hold) and 72°C with a 1 s hold, resulting in a 1.93 s cycle time. Two μM KlenTaq polymerase and 8 μM primers were used. In both cases, amplification curves of dilutions (top) and calibration curves for quantification (bottom) reveal apparent single copy sensitivity and PCR efficiencies > 90%. This figure is modified from a prior publication [15].

Figure 3 details the sensitivity and PCR efficiency obtained for two different PCR products, the 45 bp fragment of Figure 1, and a different single copy human genomic DNA fragment of 102 bp. The 102 bp product required about twice the cycle time of the 45 bp fragment, correlating loosely with their length. Amplification curves were obtained down to an average of 1.5 duplex copies per reaction, suggesting the ability to amplify even a single copy of DNA, while no template controls remained negative. The log‐linear standard curves for quantification had a correlation coefficient of 0.99, and both targets had calculated PCR efficiencies of > 90%.

**FIGURE 4:**
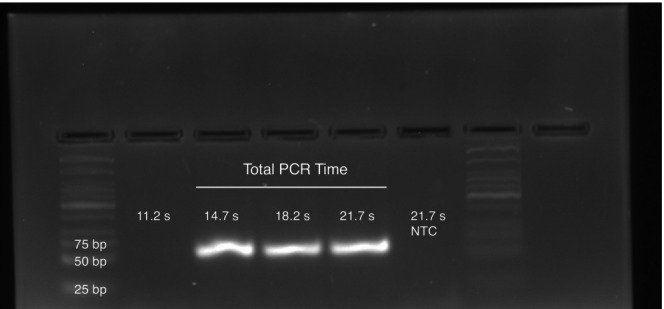
Extreme PCR in less than 15 s [15]. A 60 bp fragment surrounding an A > G variant in *AKAP10* (rs#203462) was amplified with primers GCTTGGAAGATTGCTAAAATGATAGTCAGTG and TTGACATACTGAGCCTGCTGCATAA in a 1 μL volume within a 23 gauge steel needle. Temperature cycling was between a 94°C water bath for 70 ms and a 60°C water bath for 100–400 ms. After 35 cycles in the presence of 5 mM Mg(Cl)_2_, 20 μM primers, and 8 μM KlenTaq polymerase, strong bands on gel electrophoresis were present down to 0.42 s cycle times (35 cycles in 14.7 s). This figure is modified from the supporting information of a prior publication (fig S7 of clinchem.2014.228304–2—doc file) [15].

The fastest PCR cycle time reported is 0.42 s (Table S1) [15]. A 60 bp fragment of the human genomic *AKAP10* gene was amplified in a 1 μL volume by extreme PCR in 14.7 s, giving a strong fluorescence band on an agarose gel (Figure 4). Shorter times were not successful. Very high concentrations of polymerase (8 μM) and primers (20 μM) were used. To achieve temperature cycles of less than 2 s, PCR samples in capillary tubes were physically transferred between water baths by stepper motors. A mock sample with a miniature thermocouple in water was included to monitor the temperature, and fluorescence was followed in real‐time. Systems with either two [15] or three [16] water baths were developed for either two (combined annealing and extension) or three (separate annealing, extension, and denaturation) temperature PCR. Since the original publication in 2015, other groups have used high polymerase and primer concentrations with cycle times in the 1–4 s range, including multiple groups using flow‐through microfluidics [17, 18, 19, 20, 21]. It remains somewhat ironic that the fastest system to date is not a high technology microfluidic device, but simple physical translation of tubes between water baths, reminiscent of the manual processing of samples before temperature cycling instruments became available.

Although both product denaturation and primer annealing in PCR have kinetic limits [22], faster denaturation can be achieved by increasing the denaturation temperature, and higher primer concentrations can shorten the required annealing times. The limiting factor is then the time required for polymerase extension. It is no coincidence that short PCR products can be successfully amplified faster than long products. Increasing the polymerase concentration helps to ensure polymerase binding, but extension rates depend on the temperature, the specific polymerase, the identity of the bases extended, the Mg^++^ concentration, and other environmental factors. Stopped‐flow studies reveal that secondary structures, monovalent cations (i.e., K^+^), fluorescent dyes, and probes all slow down extension rates [23, 24]. Greater understanding of the factors that affect extension rates, as well as the availability of faster polymerases in the future, should enable further progress in shortening the times of extreme PCR. However, the biochemical limits of extreme PCR are not relevant for PCR performed on conventional instruments that are limited by their ability to accurately change temperature quickly. Commercial PCR instruments have become marginally faster over the past few decades, and PCR speeds can be increased by limiting holding times [25], and/or by lowering the temperature cycling span by using high annealing primers and keeping the product melting temperature (Tm) as low as possible [26].

**FIGURE 5:**
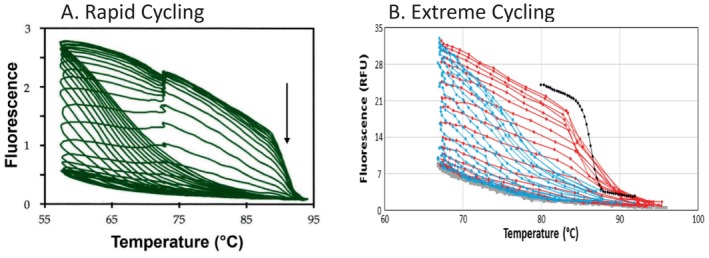
Fluorescence vs. temperature plots to monitor hybridization during rapid and extreme PCR. (A) Rapid cycle PCR was performed with 0.5 μM primers GCTCACTCAGTGTGGCAAAG and GGTTGGCCAATCTACTCCCAGG to amplify a 536 bp fragment of the human beta‐globin gene from genomic DNA using 3‐temperature cycling of 61°C (no hold), 72°C for 15 s, and 95°C (no hold), resulting in a 27 s cycle time [27]. Reagents were the same as in Figure 1, except that a 1:10,000 dilution of SYBR Green I was used to monitor PCR instead of LCGreen Plus and fluorescence was monitored every 200 ms on a prototype capillary LightCycler. (B) Extreme PCR was performed with 0.72 μM KlenTaq polymerase and 5 μM of primers GCGTGGAACCTTTTCGGCTCCT and GCTGCGAGCAAAACAAGCGGCTA, and 1× LCGreen Plus, amplifying a 79 bp fragment from 5300 copies/μL of a synthetic double stranded template of the hepatitis B sequence. Two‐temperature cycling (68°C for 0.2 s and 95°C with no hold) resulted in 1.05 s cycles on a prototype microfluidic instrument with fluorescence acquired 30 times each s [18]. At the end of PCR, a higher resolution melting curve was obtained at 8°C/s (black). In both panels, the expected decrease in fluorescence with increasing temperature is overlaid against: (1) increasing fluorescence each cycle, (2) a sharp drop in fluorescence when heating through the Tm of products, (3) an increase in fluorescence while cooling towards the annealing temperature from product re‐annealing, and (4) a fluorescence increase at the extension temperature with 3‐temperature cycling. The figure is modified from prior publications [18, 27].

Rapid cycling reduced the time required for PCR by an order of magnitude, while extreme PCR provided another 30‐fold decrease. Comparing the 3‐h procedure of original PCR with 30 s extreme PCR is a 360‐fold difference. Although the cycling times may have changed, the process has not. Figure 5 compares rapid to extreme cycling on fluorescence vs. temperature plots that visualize product hybridization continuously throughout PCR. Each data loop is one temperature cycle. The rapid cycle data (Figure 5A) was collected in 1996 with SBYR green I as dye [27] using 3‐temperature cycling on a capillary LightCycler. On approach to the annealing temperature, fluorescence increases, particularly at later cycles when product re‐annealing competes with primer annealing. At the extension temperature, fluorescence increases as extension occurs and more double‐stranded DNA is made. On approach to the denaturation temperature, there is a sharp drop in fluorescence as the product melts at its Tm (arrow). The extreme PCR data in Figure 5B was collected in 2019 with LCGreen Plus dye and 2‐temperature cycling on a microfluidic instrument [18]. The heating (red) and cooling (blue) phases are overlaid on an endpoint melting curve (black). Although there is a 27‐fold difference in cycle time and the targets and dyes are different, the product hybridization status that drives PCR is similar. The drop in fluorescence that occurs with DNA denaturation turns out to be a very useful analytical tool characteristic of what has been amplified.

## 4 Analysis

The reason to amplify targeted regions by PCR is to make detection, quantification, and characterization easier. Real‐time PCR does an admirable job of detection and quantification, and in some cases, further analysis may not be necessary. However, further characterization is often performed by size separation on gels, melting curve analysis, or sequencing. Sequencing provides the most information, and gel analysis is simple to perform, but both methods require processing, additional equipment, and cannot be performed on the time scale of interest here. In contrast, melting analysis can be performed immediately after PCR on the same instrument used for real‐time PCR without additional manipulation and can be acquired in seconds.

### 4.1 DNA Melting Analysis

Melting is a fundamental property of DNA that can be monitored by dyes that fluoresce in the presence of double‐stranded DNA, but not with single‐stranded DNA or nucleotides. In contrast to gels that measure product size, melting analysis characterizes products by their melting curves. If a product melts in a single transition, the temperature at which half the DNA is melted is referred to as the melting temperature, or Tm. Most small PCR products (< 100 bp) melt in a single transition. However, many larger PCR products melt in multiple transitions because the distribution of the more stable GC nucleotide pairs is not random. Regions with high AT content melt first, while GC‐rich regions melt later, producing a more complex melting curve that further characterizes the product. A free web application (uMelt, http://www.dna‐utah.org) predicts amplicon melting curves with multiple domains, a useful tool for verifying the identity of amplified products. Melting analysis, particularly when performed at high resolution (high resolution melting or HRM), has many useful applications including genotyping of single base variants, heterozygote scanning, methylation assessment, copy number determination, and verification of sequence identity. DNA melting analysis has been recently reviewed [28].

**FIGURE 6:**
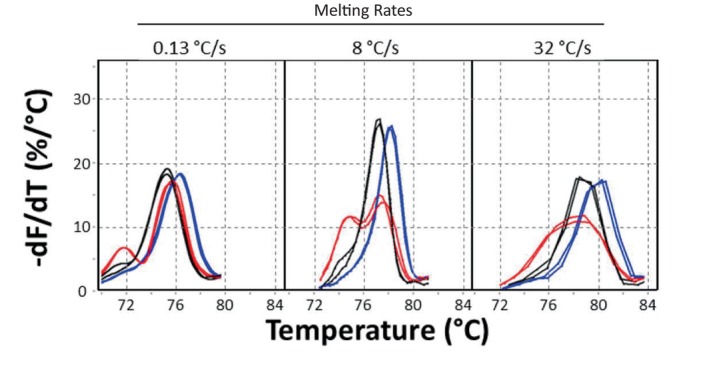
High speed melting analysis after amplification on a prototype microfluidic instrument [31]. Primers GGAGGAGCTGACCAGTGAA and AAGAACGAAGACTTCAAAGACACTT surrounding an A > C variant of *MTHFR* were used to amplify a 46 bp product from 6400 copies/μL of genomic DNA. The composition of amplification reagents and details of the thermal cycling can be found elsewhere [31]. After PCR, melting was performed from 65°C to 95°C at either 0.13, 8, or 32°C/s, measuring the fluorescence of LCGreen Plus with a camera at a frame rate of 30/s. The normalized negative derivative melting curves are shown at each melting rate for wild type (AA, black), homozygous variant (CC, blue) and heterozygous (AC, red). Although 8°C/s gives the best resolution, genotyping is still clear at 32°C/s. The jagged curves at 32°C are a result of the limited frame rate of the camera. This figure is modified from a prior publication [31].

Because melting analysis is simple to implement, low‐cost, and useful, it is incorporated into almost all real‐time PCR instruments. The easiest way for manufacturers to obtain high‐quality melting data is to slow down the rate of temperature change to improve inter‐ and intra‐sample temperature homogeneity. As a result, melting curve acquisition typically takes 15–60 min on most instruments. However, if intra‐sample temperature homogeneity is controlled, melting curve acquisition can be much faster. Working with μL volumes in capillary tubes surrounded by a metal ingot heated by a nichrome coil with melting rates between 0.01°C/s and 0.64°C/s revealed that faster rates were better for detecting heterozygotes [29]. Even smaller nL volumes in microfluidic channels are more responsive and homogeneous, with one report achieving melting in less than 7 s [30]. Even faster speeds were obtained on a microfluidic prototype where rates between 0.13°C and 32°C/s were compared [31]. The best heterozygote signal was detected at 8°C/s, and single base genotyping was still possible at 32°C/s (Figure 6). As melting speeds increased, Tms increased and derivative peak heights became narrower and taller, improving discrimination between genotypes. For small amplicons, both effects are predicted by the underlying nonequilibrium kinetic processes of duplex association and melting [22]. Fewer genotyping errors were made at faster speeds, and the optimal rate was 8°C–16°C/s, limited by the frame rate of the camera [31]. No genotyping errors were made even at 32°C/s. Since the span of temperatures necessary for melting curve analysis is about 32°C, melting curve acquisition can occur in 1–4 s. Like PCR, the speed of melting analysis is typically limited by the instrumentation, not by biochemistry. Extreme PCR followed by high‐speed melting has been completed in 52 s [18].

## 5 Conclusions

Sample preparation, real‐time PCR, and melting analysis can each occur in seconds. Put together, molecular diagnostics from sample to answer in less than 1 min are feasible. For DNA, alkaline lysis of whole blood provides amplifiable template that can be directly added to PCR mixtures. Extreme PCR in 30 s and high‐speed melting analysis in 5 s can be achieved with microfluidics. Starting from a finger prick, blood can be manually processed and genomic targets amplified in less than 1 min (Supporting Information Video). Automation of the process would be even faster and more reliable.

The methods and instruments highlighted here are not currently commercially available. However, progress is being made. A commercial instrument for 2‐min PCR is available (https://www.nextgenpcr.com), although real‐time PCR, melting analysis, and integrated sample preparation are missing. A 5‐min real‐time PCR instrument is also available (https://www.star‐array.com), and the integration of sample preparation with a 30‐min turn‐around time is under development. Perhaps most impressive is a CLIA‐Waived syndromic test (https://www.biomerieux.com) that analyzes 15 respiratory targets in 15 min that includes sample preparation, reverse transcription, a first round of multiplex PCR, a second round of nested PCR, and melting analysis for a diagnostic result.

The time for “while‐you‐wait” molecular diagnostics in seconds will come, but we are not there yet. Isothermal methods have simplified the required instrumentation, but their analysis times do not approach those possible with PCR [32]. For example, although loop‐mediated isothermal amplification (LAMP) can detect highly positive results in < 10 min, typically 30 min are needed to confirm negative samples [33]. Similarly, the fastest recombinase polymerase amplification (RPA) assays can turn positive in about 3 min, but at least 7 min are required to confirm negatives [34]. These times are too long to be feasible for, say, respiratory pathogen screening at airports. When you must wait for the results, every second counts, and only PCR can provide results in under a minute. Experimental evidence of the utility of such rapid molecular testing remains to be demonstrated.

## Author Contributions

C.T.W. wrote the manuscript and planned the experiments. L.Z., F.Y., and A.M. generated and interpreted data, and A.C. and N.K. performed literature review and edited the manuscript.

## Ethics Statement

The research reported here is appropriately cited and the writing is original without use of generative AI.

## Consent

The authors have nothing to report.

## Conflicts of Interest

C.T.W. and L.Z. receive royalties from the University of Utah for patents licensed to Biofire/bioMerieux. C.T.W. and N.K. are consultants for Co‐Diagnostics, Megarobo USA and Magic Lifescience, and have equity interests in Crestwood Technology and Faro Molecular. C.T.W. is a consultant for Nusantics, Inbiome, Pathogen DX, and Scope Microfluidics.

## Supporting information


**Data S1.** Supporting Information.

**Table S1.** Characteristics of studies with reported PCR cycle times of less than 10 s.

## Data Availability

The data that support the findings of this study are available from the corresponding author upon reasonable request.
