# Supplementary material for: Making Molecular Diagnostics Faster
Source: Int J Lab Hematol. 2025 Apr 22;48(2):272–80. doi: 10.1111/ijlh.14487 (PMC12353295; doi:10.1111/ijlh.14487)

## Slide 1
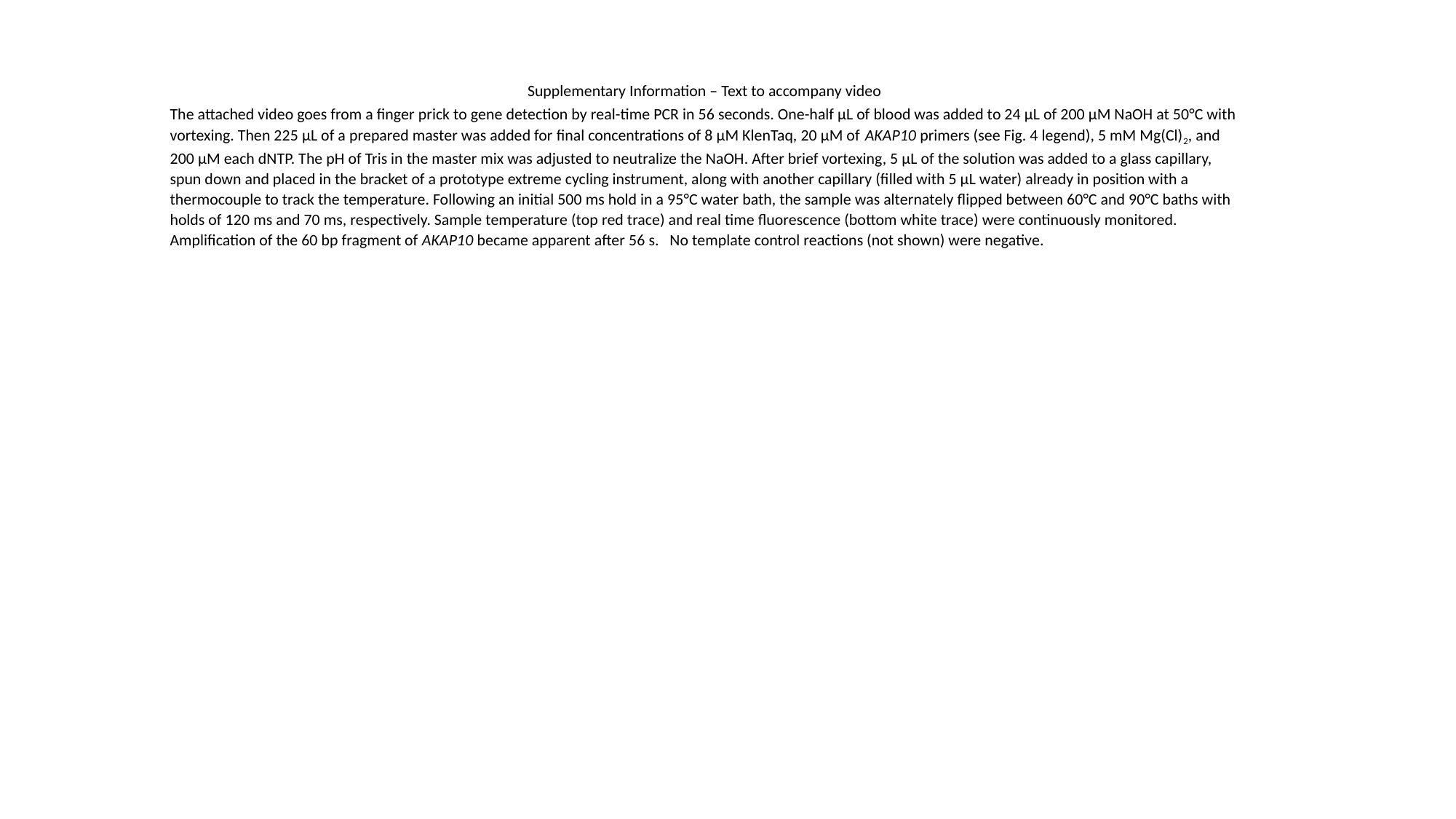

Supplementary Information – Text to accompany video
The attached video goes from a finger prick to gene detection by real-time PCR in 56 seconds. One-half µL of blood was added to 24 µL of 200 µM NaOH at 50°C with vortexing. Then 225 µL of a prepared master was added for final concentrations of 8 µM KlenTaq, 20 µM of AKAP10 primers (see Fig. 4 legend), 5 mM Mg(Cl)2, and 200 µM each dNTP. The pH of Tris in the master mix was adjusted to neutralize the NaOH. After brief vortexing, 5 µL of the solution was added to a glass capillary, spun down and placed in the bracket of a prototype extreme cycling instrument, along with another capillary (filled with 5 µL water) already in position with a thermocouple to track the temperature. Following an initial 500 ms hold in a 95°C water bath, the sample was alternately flipped between 60°C and 90°C baths with holds of 120 ms and 70 ms, respectively. Sample temperature (top red trace) and real time fluorescence (bottom white trace) were continuously monitored. Amplification of the 60 bp fragment of AKAP10 became apparent after 56 s. No template control reactions (not shown) were negative.

## Slide 2
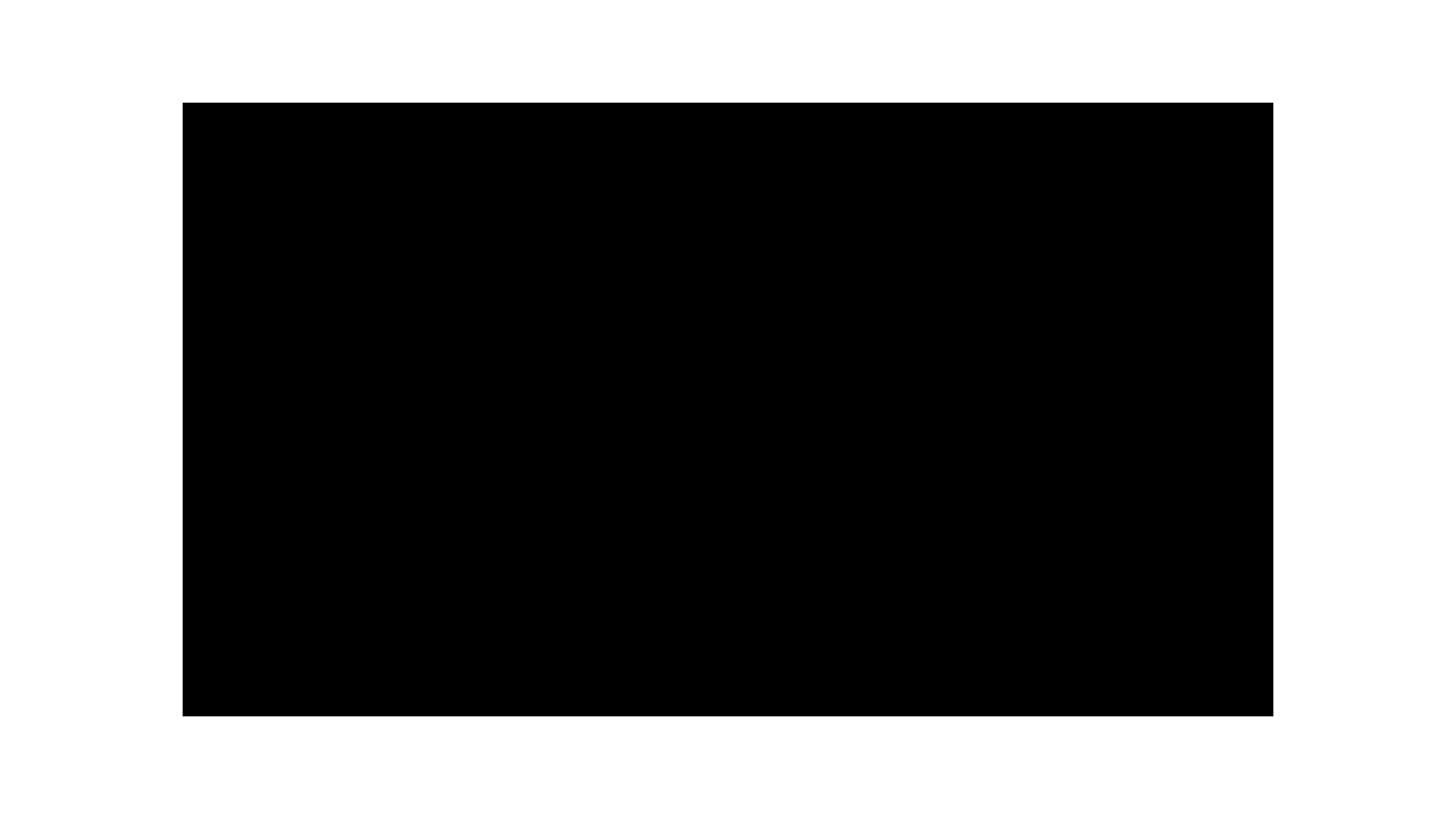

Supplement: Supplementary file 1 — Data S1. Supporting Information. [file IJLH-48-272-s002.pptx]
