# Supplementary material for: Making Molecular Diagnostics Faster
Source: Int J Lab Hematol. 2025 Apr 22;48(2):272–80. doi: 10.1111/ijlh.14487 (PMC12353295; doi:10.1111/ijlh.14487)
Supplement: Supplementary file 2 — Table S1. Characteristics of studies with reported PCR cycle times of less than 10 s. [file IJLH-48-272-s001.docx]

Supporting Information – Table S1. Characteristics of studies with reported PCR cycle times of less than 10 seconds

| **Fastest PCR Reported (s/cycle)** | **System Design** | **Heating Method** | **Cooling Method** | **Template** | **Template Concentration**  **(copies/µL)** | **Product Length (bp)** | **Polymerase** | **Polymerase Concentration**  **(nM) *** | **Primer Concentration (nM)** | **Negative Control Used?** | **Assessment of Amplification** | **Quality of Amplification** | **Group** |
| --- | --- | --- | --- | --- | --- | --- | --- | --- | --- | --- | --- | --- | --- |
|  |  |  |  |  |  |  |  |  |  |  |  |  |  |
| 0.42 | Capillary tube moved between 2 water baths via motor | Water | Water | Human | 3,000 | 60 | Klentaq | 8,000 | 20,000 | Yes | Real-time monitoring | Robust | [2015 Farrar and Wittwer](http://www.ncbi.nlm.nih.gov/pubmed/25320377) |
| 1.05 | Flow-through (oscillating) | Thin-film platinum resistor within microchannel | Passive cooling  (heat sink) | Synthetic | 5,000 | 70 | Klentaq | 3,500 | 5,000 | Yes  (not shown) | Melting curve analysis | Robust | [2019 Myrick et al.](https://pubmed.ncbi.nlm.nih.gov/30459167/) |
| 1.06 | Flow-through | Copper block | Copper block | Synthetic | 2,100 | 69 | Klentaq | 2,000 | 5,000 | Yes | Melting curve analysis | Moderate | [2017 Trauba and Wittwer](https://www.researchgate.net/publication/317000671_Microfluidic_Extreme_PCR_1_minute_DNA_Amplification_in_a_Thin_Film_Disposable) |
| 1.8 | Radiative using Au nanoparticles | Radiative (IR laser) | Chilled airflow | Plasmid | NA | 79? | KAPA2G | 4 | 500 | Yes  (low signal) | Endpoint fluorescence | Weak | [2017 Roche et al.](https://pubmed.ncbi.nlm.nih.gov/28443837/) |
| 2 | Flow-through | Copper block | Copper block | Human | 1,500 | 102 | Klentaq | 64 | 2,000 | Yes | Real-time monitoring & melting | Moderate | [2018 Jafek et al.](https://pubs.acs.org/doi/abs/10.1021/acs.analchem.7b05176) |
| 2.6 | Pressurized gas and capillaries | Forced air convection | Forced air convection | Bacteria | 360 | 85 | Taq | 100 | 500 | No | Electrophoresis gel | Weak | [2004 Whitney](http://digitalcommons.unl.edu/dissertations/AAI3131568/) |
| 2.7 | Capilary tubes in gallium eutectic | Peltier | Peltier | Bacteria | NA | 85 | KOD | NA | 500 | No | Electrophoresis gel | Weak | [2010 Maltezos et al.](http://scitation.aip.org/content/aip/journal/apl/97/26/10.1063/1.3530452) |
| 3 | Flow-through | Aluminium | Passive cooling (aluminium) | Bacteria | 1 x 10^4^ | 134 | Ex Taq | 13 | 200 | Yes  (low signal) | Real-time monitoring | Weak | [2011 Fuchiwaki et al.](http://www.ncbi.nlm.nih.gov/pubmed/21778045) |
| 3 | Flexible capillary moved between 2 water baths via motor | Water | Water | Soybean | 3x10^-2^ - 300 | 165 | SpeedSTAR | 54 | 400 | Yes | Electrophoresis gel & imaging | Moderate | [2018 Wang et al.](https://pubmed.ncbi.nlm.nih.gov/29333552/) |
| 3.7 | Microfluidic chip | Peltier | Peltier with | Human cDNA | 3,750 | 89 | Taq (HotStart) | 50 | NA (no primer info) | Yes  (not shown) | Electrophoresis gel | Weak | [2019 Lee et al.](https://pubmed.ncbi.nlm.nih.gov/30553102/) |
| 4 | Flow-through | Polyimide film heaters | Passive cooling | Leukemia cell line K562 | 670 | 151 | KlenTaq | 6,000 | 3000 | No | Electrophoresis gel | Robust | [2022 Lin et al.](https://pubmed.ncbi.nlm.nih.gov/35624604/) |
| 4 | Droplet PCR (in oli) | Radiative (IR laser) | Passive cooling | Bacteria | 33 ~ 100 | 72 | KOD | NA | NA | No | Real-time monitoring | Moderate | [2008 Terazono et al.](http://iopscience.iop.org/article/10.1143/JJAP.47.5212/meta) |
| 4.3 | Flow-through | Aluminium | Aluminium | Bacteria | 125 | Multiplex  197 & 311 | SpeedSTAR | 54 | 400 | Yes  (not shown) | Electrophoresis | Weak | [2019 Li et al.](https://pubmed.ncbi.nlm.nih.gov/31273367/) |

Table S1 (continued)

| **Fastest PCR Reported (s/cycle)** | **System Design** | **Heating Method** | **Cooling Method** | **Template** | **Template Concentration**  **(copies/µL)** | **Product Length (bp)** | **Polymerase** | **Polymerase Concentration**  **(nM) *** | **Primer Concentration (nM)** | **Negative Control Used?** | **Assessment of Amplification** | **Quality of Amplification** | **Group** |
| --- | --- | --- | --- | --- | --- | --- | --- | --- | --- | --- | --- | --- | --- |
|  |  |  |  |  |  |  |  |  |  |  |  |  |  |
| 4.6 | Convective heat transfer through porous copper media with resistive heating | Water | Water | Bacteria | 1.9 x 10^5^ | 160 | KAPA2G | 9 | 400 | Yes  (not shown) | Electrophoresis gel | Moderate | [2011 Wheeler et al.](https://pubs.rsc.org/en/content/articlelanding/2011/an/c1an15365j#:~:text=This%20method%20of%20rapid%20heat,breakthrough%20speeds%20on%20our%20system.) |
| 4.8 | Silicon chip on Peltier | Peltier | Peltier | Bacteria | 4.2 - 4,200 | 78 | KAPA2G | 370 | 20,000 | Yes | Real-time monitoring & melting | Robust | [2019 Cai et al.](https://pubmed.ncbi.nlm.nih.gov/30348381/) |
| 5 | Capillary tube moved between 2 water baths | Water | Water | Maize | NA | 195 | Taq | 300 | 2,000 | Yes | Electrophoresis gel | Moderate | [2019 Gao et al.](https://pubmed.ncbi.nlm.nih.gov/30611487/) |
| 5 | Digital droplet PCR between two water baths | Water | Water | Bacteria | 10^-3^ – 1,000 | 192 | SpeedSTAR | 86 | 400 | Yes | Digital endpoint fluorescence | Moderate | [2023 Wang et al.](https://pubmed.ncbi.nlm.nih.gov/37887103/) |
| 5.2 | Flow-through  (polycarbonate) | Resistance film heaters | Passive cooling | Lambda phage | 2 x 10^8^ | 500 | Taq | 14 | 200 | No | Electrophoresis gel | Weak | [2004 Hashimoto et al.](http://www.ncbi.nlm.nih.gov/pubmed/15570378) |
| 5.3 | Water pumped against aluminum plate with 1µL droplets | Aluminium | Aluminium | Bacteria | 2.2 x 10^6^ | 72 | KOD | 24 | 250 | Yes | Real-time monitoring | Robust | [2010 Terazono et al.](http://iopscience.iop.org/article/10.1143/JJAP.49.06GM05/meta) |
| 5.6 | Droplet shuttled between zones using electrowetting | ITO resistive heating | Passive cooling | Plasmid | 1.7 x 10^5^ | 63 | KAPA2G | 22 | 500 | Yes | Real-time monitoring | Moderate | [2023 Wan et al.](https://pubmed.ncbi.nlm.nih.gov/37797533/) |
| 5.7 | Flow-through | Aluminium | Aluminium | Bacteria | NA | Multiplex 197 & 316 | SpeedSTAR | 1 | 200 | Yes  (not shown) | Electrophoresis | Moderate | [2021 Li et al.](https://pubmed.ncbi.nlm.nih.gov/34190300/) |
| 6.3 | Flow-through | Aluminium | Aluminium | Plasmid | 400 | NA | Ex Taq | 13 | 400 | Yes  (low signal) | Endpoint fluorescence | Moderate | [2011 Fuchiwaki et al.](http://www.ncbi.nlm.nih.gov/pubmed/21415501) |
| 6.5 | Photonic heating on a chip with TiN nanorings | Radiative  (IR LED) | Passive cooling | Lambda phage | NA | 100 | Taq | NA | 400 | No | Electrophoresis gel | Weak | [2024 Kim et al](https://pubmed.ncbi.nlm.nih.gov/39086075/). |
| 6.6 | Flow-through  (silica chip) | Copper block | Copper block | PCR product | 1 x 10^7^ | 176 | Taq | 50 | 1,000 | Yes | Electrophoresis gel | Weak | [1998 Kopp et al](http://www.ncbi.nlm.nih.gov/pubmed/9582111). |
| 6.6 | Gold nanoislands on glass nanopillar arrays | Radiative (White LED) | Passive cooling | Lambda phage | 9,000 | 98 | Taq | 5 | 9,000 | Yes | Real-time monitoring | Robust | [2021 Kang et al.](https://pubmed.ncbi.nlm.nih.gov/34008961/) |
| 6.7 | Primer-immobilized hydrogels containing graphene oxide | Radiative (NIR LED) | Passive cooling | Synthetic | 1 x 10^7^ | 59 | Taq | NA | NA | Yes | Real-time monitoring | Robust | [2022 Kim et al.](https://pubs.acs.org/doi/10.1021/acsnano.2c07017) |

Table S1 (continued)

| **Fastest PCR Reported (s/cycle)** | **System Design** | **Heating Method** | **Cooling Method** | **Template** | **Template Concentration**  **(copies/µL)** | **Product Length (bp)** | **Polymerase** | **Polymerase Concentration**  **(nM) *** | **Primer Concentration (nM)** | **Negative Control Used?** | **Assessment of Amplification** | **Quality of Amplification** | **Group** |
| --- | --- | --- | --- | --- | --- | --- | --- | --- | --- | --- | --- | --- | --- |
|  |  |  |  |  |  |  |  |  |  |  |  |  |  |
| 7 | Gold nanoislands on glass nanopillar arrays | Radiative (White LED) | Passive cooling | Lambda phage | 1.8 x 10^6^ | 98 | Taq | 5 | 9,000 | Yes | Electrophoresis gel | Robust | [2020 Lee et al.](https://pubs.acs.org/doi/10.1021/acsami.9b23591) |
| 7.5 | Silicon chip | Peltier | Peltier | Pseudo virus | NA | 116 | Kapa2G | 40 | 360 | Yes | Real-time monitoring | Moderate | [2022 Zhang et al.](https://pubmed.ncbi.nlm.nih.gov/36832000/) |
| 8.5 | Micromachined cantilever terminated in a disc | Thin silicon  film heater | Passive cooling | Mouse cDNA | NA | 82 | AmpliTaq | NA | 900 | No | Real-time monitoring | Robust | [2006 Neuzil et al.](http://www.ncbi.nlm.nih.gov/pmc/articles/PMC1904101/) |
| 9 | Flow-through (circular chip) | Copper block | Passive cooling | Lambda phage | 1.8 x 10^7^ | 500 | Taq | 5 | 1,000 | Yes | Electrophoresis gel | Moderate | [2007 Sun et al.](http://www.ncbi.nlm.nih.gov/pubmed/17653343) |
| 9 | Droplets in oil | Radiative  (IR laser) | Passive cooling | Human 18S rDNA | 1 x 10^6^ | 187 | AmpliTaq | NA | 900 | No | Real-time monitoring | Robust | [2009 Kim et al](http://www.ncbi.nlm.nih.gov/pubmed/19129891). |
| 9.8 | Plasmonic PCR with Au film | Radiative  (Blue LED) | Passive cooling | Human cDNA | 2,000 | 116 | KAPA2G | 1,100 | 1,000 | No | Electrophoresis gel | Weak | [2015 Son et al.](https://pubmed.ncbi.nlm.nih.gov/26592501/) |
| < 10 | Plasmonic PCR with Au film | Radiative  (Blue LED) | Forced air convection | Lambda phage | 1. 8 x 10^6^ | 98 | Taq | 5 | 900 | No | Electrophoresis gel | Weak | [2015 Son et al.](https://www.nature.com/articles/lsa201553) |
| 10 | Capillary tube moved between 2 water baths | Water | Water | Bacteria | NA | Multiplex  123 & 284 | ExTaq | 380 | 2,000 | Yes | Electrophoresis gel | Robust | [2018 Tian et al.](https://www.sciencedirect.com/science/article/abs/pii/S0925400518300170) |
| 20 | Air-cycling system using thin glass capillary tubes | Forced air convection | Forced air convection | Human | 1,500 | 536 | Taq | 16 | 500 | Yes  (not shown) | Electrophoresis gel | Robust | [1990 Wittwer et al.](http://www.ncbi.nlm.nih.gov/pubmed/2363506) |

*Notes:* Ordered according to the fastest successful cycle time reported in the paper, whether through direct citation, or dividing the reported total time of amplification by the number of cycles. Additional time before or after amplification (such as incubation steps) were not included. The 1990 report of 20 second cycles is listed at the end. "Negative Control Used?" asks whether an appropriate no-template control (NTC) was used with the fastest protocol. “Yes” means that it was, and the target signal was not observed. “No” means that an NTC was not mentioned, and the reaction specificity is uncertain. “Yes (low signal)” means that fluorescence was detected in the NTC, but amplification was considered successful for the target if the fluorescence was above a threshold. "Flow Through" is defined as a microfluidic system that moves DNA solution through different zones of temperature, such as by serpentine channels

Abbreviations: Au (gold); Cq (quantification cycle; replaces Ct or equivalent); IR (infrared); ITO (indium tin oxide); LED (light-emitting diode); NA (information not available); NIR (near infrared); TiN (titanium nitride)

*Polymerases concentrations were either measured by absorbance at 280 nm (if pure enzyme was available) or converted from activity units according to Table 1 of Montgomery JL, Rejali N, Wittwer CT. Stopped-flow DNA polymerase assay by continuous monitoring of dNTP incorporation by fluorescence. Anal Biochem. 2013;441:133-139. (PMID: 23872003, DOI: [10.1016/j.ab.2013.07.008](https://doi.org/10.1016/j.ab.2013.07.008)). For heat stable enzymes like those used in PCR, concentrations are easier to compare than unit definitions that are often variable. Polymerase enzymes (vendors): AmpliTaq (Thermo Fisher); ExTaq (Takara bio); KAPA2G (KapaBiosystems); KlenTaq (DNA Polymerase Technology); KOD (Toyobo); SpeedSTAR (TakaraBio); Taq (multiple sources).
